# Integrated protocol for the prevention and treatment of skin ulcers in patients with end-stage renal disease

**DOI:** 10.1016/j.mex.2023.102482

**Published:** 2023-11-10

**Authors:** Stefano Mancin, Beatrice Mazzoleni, Francesco Reggiani, Marta Calatroni, Elena Alterchi, Daniela Donizzetti, Silvia Finazzi, Fanny Soekeland, Marco Sguanci, Salvatore Badalamenti

**Affiliations:** a Department of Biomedicine and Prevention, University of Rome “Tor Vergata”, Rome, Italy; b Nephrology and Dialysis Unit IRCCS Humanitas Research Hospital, Rozzano, Milan, Italy; c Department of Biomedical Sciences, Humanitas University, Pieve Emanuele, Milan, Italy; d School of Health Professions, University of Applied Sciences, Bern, Switzerland; e Department of Medicine and Surgery, Research Unit of Nursing Science, Università Campus Bio-Medico di Roma, Rome, Italy

**Keywords:** Chronic kidney disease, Chronic ulcers, Wound care, Protocol, Dialysis treatment, Integrated protocol for the prevention and treatment of skin ulcers in patients with end-stage renal disease

## Abstract

Chronic Kidney Disease (CKD) is an escalating global health concern, affecting more than 10 % of the general population worldwide, amounting to over 800 million individuals. One of its major complications for patients is the high prevalence of skin ulcers . This study aims to develop a protocol for ulcer management within the context of a hospital-based dialysis center. The success of this strategy is deeply rooted in the collaboration of a multidisciplinary team, continually enriched by specialist training. The clinical nurse specialist (CNS) in wound care plays a pivotal role in this approach. By employing a systematic methodology, the protocol is tailored to emphasize holistic care for patients diagnosed with end-stage renal disease undergoing hemodialysis. It accentuates the significance of proactive prevention, in-depth patient education, and the immediate identification of early wound signs. The research underscores the necessity to further weave in specialized training for ulcer care, ensuring each hospital visit is maximized for efficiency and effectiveness. Central to this protocol is the understanding that CKD is a growing concern, that the optimal management of ulcers relies heavily on multidisciplinary collaboration, and that an emphasis on prevention, patient education, and timely wound recognition is crucial to enhance patient care and experience.

## Graphical abstract

**Figure:**
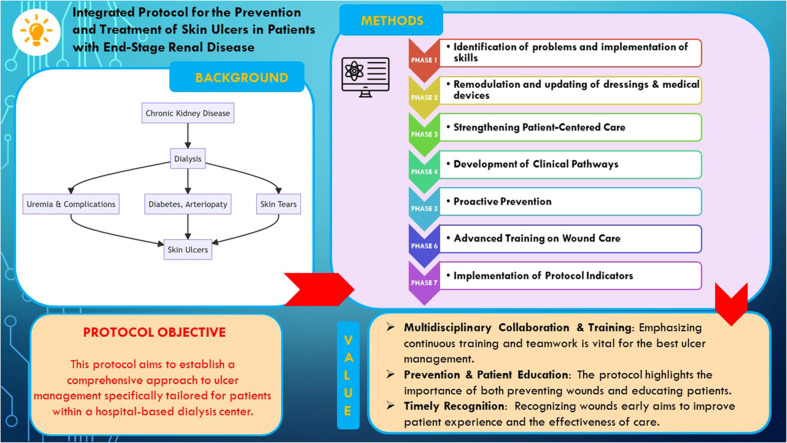

Specifications table

**Table:** 

Subject area:	Medicine and Dentistry Medicine and Dentistry
More specific subject area:	Wound Care
Name of your protocol:	Evidence Based Methodology protocol
Reagents/tools:	Advanced dressings
Experimental design:	Retrospective Observational Study
Trial registration:	The study received approval from the Institutional Review Board of IRCCS Humanitas Rozzano Milan.
Ethics:	The study adhered to the guidelines of the Declaration of Helsinki. All participants provided informed consent to participate in the study.
Value of the Protocol:	• **Patient-Centered Holistic Care**: The protocol emphasizes a holistic approach to wound care specifically tailored for CKD patients undergoing hemodialysis. • **Specialized Clinical Approach**: With the integration of specialized clinical expertise in wound care and the systematic methodology employed, the protocol ensures that ulcer management aligns with the latest scientific evidence and best practices, upholding high standards of care. • **Proactive Prevention and Education**: By prioritizing proactive prevention and comprehensive patient education, the protocol aims not just to treat, but also to prevent the onset and progression of ulcers, thereby optimizing patient outcomes and experiences.

## Background

Chronic Kidney Disease (CKD) is increasingly becoming a major public health issue worldwide, affecting more than 10 % of the general population, which amounts to over 800 million individuals, and demonstrating a rising incidence in both developed and developing countries. [1]. It is characterized by a progressive loss of kidney function, and when it reaches its end stage, patients are required to undergo dialysis treatments [2]. This patient group faces an range of complications, including hypertension, anemia, electrolytic imbalances, and edema [3,4]. Additionally, factors that worsen CKD include malnutrition [5], strict dietary restrictions, and imbalances in the gut microbiota, attributed to both to the underlying pathology and the administered pharmacological treatments [6]. One of the more severe clinical conditions that burden these patients is the high prevalence of skin ulcers [7]. The etiology of these ulcers can be varied vary, including related conditions such as diabetes mellitus, arterial diseases, uremic complications [8], and nutritional deficiencies [3]. Beyond the considerable physical discomfort caused by these ulcers, they also present significant logistical challenges [9]. Patients, who are already burdened by hospital visits for dialysis treatments requiring frequent outpatient admissions, must also manage additional outpatient visits for skin ulcer care [10].

Given these complexities, the need to establish targeted protocols for ulcer management becomes paramount [10]. This aims not only to reduce complications and risks associated with ulcer care but also to optimize the patient's experience during dialysis sessions. Furthermore, it seeks to ensure that every hospital visit is as efficient and effective as possible, considering that patients also face a decline in their quality of life and physical mobility alterations [11]. Central to this landscape is the figure of the Clinical Nurse Specialist (CNS) specialized in wound care. Endowed with extensive postgraduate training and a comprehensive clinical experience, the CNS possesses specialized skills in skin ulcers management [12]. Through an evidence-based approach, the CNS plays a pivotal role in maintaining high standards of care, guiding and training the care team, and monitoring patients' clinical progress [13].

### Protocol objective

In consideration of the intricate nature of ulcer treatment in CKD patients and acknowledging the indispensable role of specialized healthcare professionals, our primary objective is to develop a detailed protocol to manage skin ulcers within a hospital-based dialysis center context. Through this protocol, we aim to integrate ulcer care into the overall patient care framework, guaranteeing safety, effectiveness, and a substantial improvement in the quality of life for our patients.

## Methods

### Protocol overview

In this research, we employed a systematic methodology to delineate a clinical protocol emphasizing wound care holistic care for patients diagnosed with end-stage renal disease undergoing hemodialysis [10]. Given the significant clinical implications of skin wounds in this specific population [14], our protocol was carefully crafted to improve wound management, simplify patient access to specialized outpatient services, and elevate the overall quality of healthcare provided [10]. The protocol predominantly underscores the significance of proactive prevention, thorough patient education, and prompt identification of initial signs of wounds, all aimed at reducing associated complications [10,15]. The protocol presented is based on an original retrospective observational study conducted over a period of three years, starting in December 2015 and concluding with database analysis in 2019 at a tertiary hospital in Northern Italy. The study received approval from the Institutional Review Board of IRCCS Humanitas Rozzano Milan, adhering to the guidelines of the Declaration of Helsinki, with all participants granting informed consent [10]. The study encompassed 110 hemodialysis patients, primarily elderly and non-self-sufficient individuals, a subgroup highly susceptible to skin wound development [14]. This condition profoundly impacts their quality of life, further compounded by challenges in accessing specialized clinics for preventative check-ups and diagnostic tests [10]. The mean age of the study cohort was 67 years, with a slight male predominance of 61 %. The sample exhibited a diabetes prevalence of 42.5 % and an arterial disease prevalence of 32 %. This research led to a significant enhancement in nursing care quality, as evidenced by a 46 % reduction in skin wound incidence. Additionally, the study promoted the refined application of knowledge by the multidisciplinary team, with nurses optimizing the use of advanced dressing devices. It's crucial to recognize that, within this protocol's presentation framework, specific implementation timeframes were not delineated, as they might vary based on the infrastructural and organizational dynamics of healthcare facilities. A tabulated depiction of this protocol is presented in Table 1.

**Table 1:** Detailed protocol phases for wound management in hemodialysis patients.

Protocol Phase	Objectives	Description
P1	Identification of problems and implementation of skills	Multidisciplinary group meetings Specialized training sessions
P2	Remodulation and updating of devices for the treatment of skin lesions	Introduction of advanced dressings Establishment of wound data archive
P3	Enhance continuity of care	Development of a specific wound assessment form for CKD patients Creation of a patient satisfaction questionnaire
P4	Facilitate the care for management of Complex Wounds	Clinical pathways for diabetic foot ulcer and vascular ischemic ulcer
P5	Preventative measures for high-risk patients	Educational programs and brochures
P6	Staff training course	Residential courses on wound care in patients with CKD
P7	Assessment and evaluation of the implemented measures	Analysis of data on the incidence of the wound, type, healing times Analysis of patient satisfaction data Economic impact analysis

### Protocol description

#### Phase 1: identification of problems and implementation of skills

**Fig. 1:**
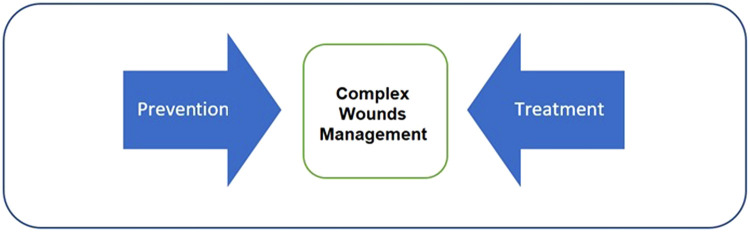
Identification and evaluation of problems.

**Legend**: The illustration identifies the initial problems that emerged during meetings between members of the multidisciplinary team in the treatment of wounds

The first phase centered on an in-depth investigation into “difficult wounds” with a specific focus on patients undergoing hemodialysis treatments. To thoroughly explore the issue and identify optimal solutions, a series of four meetings weremeticulously planned. Each of these meetings, with a maximum duration of two hours, actively involved a multidisciplinary team composed of nephrologists and nurses specialized in hemodialysis [10]. During these discussions, the primary objective was to recognize and analyze the key challenges and complications associated with the management of difficult wounds and skin ulcers during dialysis sessions [14]. Through constructive interactions and a continuous exchange of experiences among participants, two crucial aspects emerged: the need to develop an effective preventive strategy for wounds and the establishment of the most efficient practices for their treatment during dialysis sessions [10] (Fig. 1).

However, despite the significance of these findings, another challenge was identified during the analysis: a deficiency in specific skills among healthcare professionals in the treatment of difficult wounds [10,16]. This gap highlighted the fundamental need to integrate a highly specialized role into the healthcare team: the wound care nurse specialist. This professional is tasked not only to be a central point of reference for patients, providing targeted and personalized care, but also to serve as a consultant for the entire multidisciplinary team [17]. In doing so, they facilitate a synergistic connection among the various skill sets present and ensure a therapeutic approach based on the best available scientific evidence [12].The incorporation of this new role aspired to promote continuous education and implementing clinical protocols grounded in the latest research and best practices in the field [12]. Consequently, specialized training events were organized. These sessions were distinguished by being more than mere theoretical lessons; they constituted practical field training [15]. During these training sessions, healthcare personnel had the opportunity to engage directly with cutting-edge techniques, gaining hands-on experience and directly assimilating insights from real clinical cases. This practical and tangible approach allowed for the direct management of wounds in daily practice, enhancing the team's capabilities and ensuring an innovative therapeutic approach firmly rooted in scientific evidence [10].

#### Phase 2: Remodulation and updating of dressings & medical devices

The second phase was primarily dedicated to optimize the medical dressing devices used for treating skin lesions. Through a meticulous assessment of the currently utilized materials, innovative changes were rolled out to enhance their efficacy [10].

##### Advanced dressings

One of the landmark interventions at this juncture was the introduction of advanced dressings, chosen specifically based on the distinct characteristics of patients' wounds and were rooted not in randomness but in the freshest clinical guidelines and good practice recommendations [17], [18], [19], [20], [21]. The overarching objective was to ascertain that each patient was bestowed with the most contemporary and apt treatment, striking a balance between elevating care quality and cost containment.

##### Data collection and monitoring

Coinciding with the material enhancements, a structured database emerged on the horizon. This database was meticulously crafted to:

• Collect, categorize, and oversee data pertinent to challenging wounds.
• Offer a comprehensive and organized snapshot of patient conditions, fostering incessant monitoring.
• Facilitate an intricate examination of the progression of wounds over time.

This dual approach, combining the refinement of material assets with the inauguration of a digitized data repository, laid the foundational stones for a more pinpointed treatment of skin lesions. Additionally, it optimized the management of clinical data and insights [10].

#### Phase 3: Strengthening patient-centered care

During the third phase, the emphasis was placed on the customization and efficiency of the care provided to patients with skin lesions. Recognizing the significance of an individualized approach, especially for chronic patients, we dedicated efforts to create and validate a specific tools:

• a dedicated wound monitoring sheet. This “wound assessment sheet” was meticulously designed to be fully adapted to the characteristics of each individual patient. The primary objective was to ensure enhanced continuity of care, ensuring that every patient received consistent treatment and monitoring tailored to their specific needs. The creation of this document resulted from extensive collaboration and internal validation procedures within our hospital facility, with the aim of ensuring its effectiveness and reliability in the clinical context [10].
• a questionnaire designed to assess the patient's satisfaction level concerning the care received for their wounds. The introduction of this questionnaire aimed to gather direct feedback from patients, allowing us to better understand their needs and expectations and, where necessary, make adjustments to our clinical approach. The questionnaire, distributed every 12 months, was completely anonymous and optional; it evaluated the following themes: assistance from the multidisciplinary team (1), quality of care provided (2), access to dedicated clinics and specialist visits (3), patient education (4) and awareness of the “problem” of complex wounds (5). For each item, four questions were provided, measured according to a rating scale from 1 to 10, for a total of 20 questions. The threshold value to demonstrate an adequate degree of satisfaction was the achievement of an average percentage of satisfaction ≥ 85 % in the five domains investigated [10].

The introduction of this questionnaire in this phase served as a pilot tool in order to identify the level of quality of care provided and to implement possible strategies to improve the protocol; this phase represented a key step in our commitment to provide high-quality patient-centered care based on the best available scientific evidence. Through the adoption of these new tools, we have improved our ability to monitor, treat and interact with patients, guaranteeing them an optimal and personalized therapeutic path.

#### Phase 4: Development of clinical pathways for the management of complex wounds

**Fig. 2:**
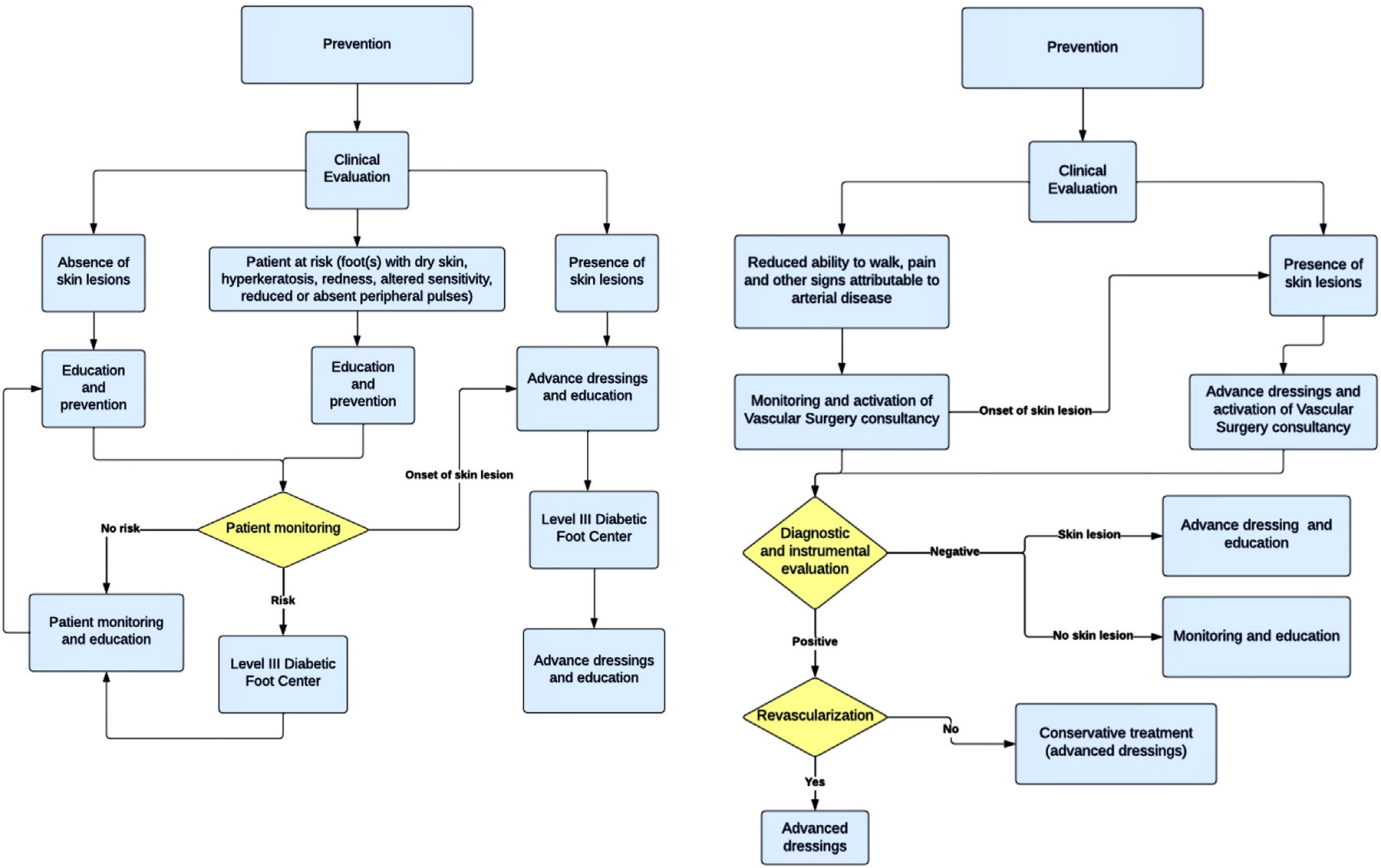
Clinical pathways for the management of complex wounds.

**Legend:** The illustration showcases, from left to right, the two Clinical Pathways developed within the protocol for the management of diabetic foot ulcers and vascular ischemic ulcers

During this phase, efforts were directed towards the establishment of clinical pathways specifically dedicated to the two types of lesions most debilitating for these patients: the diabetic foot ulcer and the vascular ischemic ulcer [4]. The primary purpose of these clinical pathways was to simplify and optimize the care provided to patients affected by these conditions. This allowed for quicker access to specific and advanced treatments, accelerating the healing process and considerably improving the prognosis [10]. The clinical pathways were developed based on available guidelines [18], [19], [20], [21], while always considering the specific services and resources available within the hospital facility. This approach allowed the creation of an integrated system [10], in which the guidelines and available resources perfectly intertwined, ensuring high-quality, timely care tailored to the specific needs of each patient. Moreover, an additional goal was to provide a clear and structured framework, crucial for preventing complications and enhancing the quality of life of patients (Fig. 2).

#### Phase 5: Proactive prevention

**Fig. 3:**
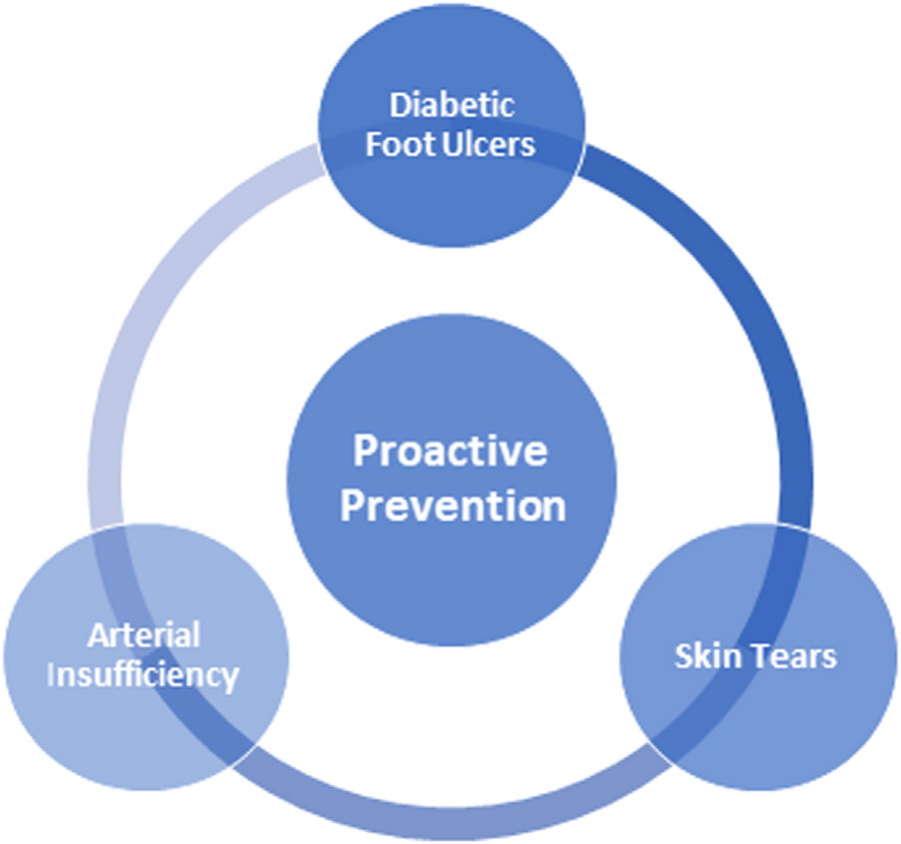
Proactive prevention for the management of complex wounds.

**Legend**: The illustration highlights the three key pathologies on which proactive prevention was centered. Using targeted brochures, the aim was to strengthen the awareness and adherence of CKD patients to preventive guidelines and therapeutic education.

During Phase 5, we undertook a significant initiative to enhance the prevention of the development of complex skin lesions [10] (Fig. 3). Recognizing that prevention is key to countering the progression of acute lesions into hard-to-heal chronic ulcers, a comprehensive information campaign was launched [14], [15], [16]. In this context, various targeted brochures were developed and distributed according to the specific needs of the patients.

• Diabetes Patients received brochures specifically formulated for the prevention of diabetic foot ulcers, as these lesions are a common complication in this group.
• Patients diagnosed with arterial insufficiency or considered at risk of developing vascular complications were provided with informative materials aimed at preventing vascular lesions [10].
• Patients at risk of skin tears. These lesions, characterized by a separation or tear in the skin often due to minor trauma or friction, are particularly problematic in elderly patients or those with skin alterations [10,22]. If not managed correctly, skin tears can rapidly degenerate into chronic and complex wounds. Therefore, brochures dedicated to the prevention of skin tears were provided to all patients, regardless of their specific risk profile

#### Phase 6: Advanced training on wound care

During this phase, the focus shifted to enhancing the skills of healthcare professionals in the prevention and management of complex skin wounds in CKD patients. To achieve this objective, we conducted two intensive residential courses, each spanning eight hours in duration [10]. Both courses were designed for members of the multidisciplinary dialysis clinic team, encompassing nurses and nephrologists.

• The first course served as foundational training, aimed at strengthening the fundamental knowledge in the care, management, and prevention of complex wounds- [16].
• The second course was specifically tailored to address the nuances of managing challenging wounds in CKD patients [14].

A pivotal component of both courses was face-to-face frontal teaching. This approach, reliant on direct interaction between instructors and participants, was complemented by a segment focused on clinical case studies. Through the analysis of these real-world cases, participants were prompted to exercise their critical thinking, applying theoretical notions in an applied context [10,16]. This experiential learning methodology allowed participants to consolidate their knowledge, tackling real-world issues, and devising appropriate solutions in a controlled environment.

To assess the training's efficacy and ensure learning objectives were met, a 20-question evaluation questionnaire was administered at the conclusion of each course. The outcomes of this questionnaire were crucial in determining participants' comprehension and, consequently, their successful completion of the course [10].

#### Phase 7: Implementation of protocol indicators

In this final phase, we proceeded to analyze and compare the data from the study period [10]. This crucial stage allowed us to quantify and assess various aspects of our clinical practice and the efficacy of the implemented protocol. The outcome indicators we considered included:

• The overall incidence of skin wounds.
• The incidence of specific types of skin wounds: diabetic ulcers, vascuopathic lesions, and skin tears.
• Healing times by the 90th day.
• Lesions that did not resolve or heal.

In addition to outcome indicators, we also evaluated efficiency indicators, which provided clear insight into the associated treatment costs and potential savings resulting from the implementation of the protocol. Furthermore, we examined the use of advanced wound dressing devices as an indicator of the quality of care provided [10,17].

Finally, as a satisfaction indicator, we analyzed the results of satisfaction questionnaires administered to patients. This allowed us to gain a deeper understanding of the patients' perceptions and opinions about the treatment and care they received. The collection, analysis, and comparison of these indicators were fundamental for a comprehensive evaluation of our protocol's efficacy. Through this process, we identified areas of success and potential sectors where further improvements might be necessary.

### Challenges and limitations

Our protocol offers numerous advantages in managing skin lesions in CKD patients undergoing hemodialysis. Nevertheless, it is essential to consider some inherent limitations and challenges faced during its implementation.

• *Specificity:* The protocol was optimized for patients with end-stage renal disease, meaning its applications might not be universally transferable to other populations or clinical conditions. This specificity, however, ensures meticulous and targeted care for the demographic in question.
• *Subjectivity in Surveys:* While every effort was made to minimize biases in data collection and interpretation, the nature of satisfaction surveys might introduce some subjectivity. This, however, authentically reflects the patient's perception regarding their care.
• *Long-Term follow-up:* Even though the original study spanned a significant period, a longer-term follow-up could provide more comprehensive insights into the sustainability and ongoing efficacy of the protocol.
• *Implementation of the use of advanced dressings:*During the protocol phases, one of the most significant challenges was the implementation of new advanced dressing aids. This challenge was addressed through residential courses aimed at the multidisciplinary team.
• *Patient and Caregiver Education*: Especially among elderly patients, imparting knowledge can be challenging. The problem was tackled using simple brochures specifically created for patient subgroups (diabetics, arteriopathics, and those at risk of skin tears).

## Conclusions

This protocol, grounded in a holistic approach to manage skin lesions in CKD patients undergoing hemodialysis, stands as a pivotal contribution to the current landscape of managing complex wounds. The emphasis on the importance of prevention, patient-centered management, and targeted education are key elements that elevate the protocol above standard approaches. The introduction of a wound care specialized nurse markes a turning point in care, underlining the significance of continuous specialist training. The analysis of various indicators permits a comprehensive evaluation of the protocol's efficacy from both a clinical and economic perspective. Despite certain limitations, the protocol presents a robust and dynamic model, which with periodic reviews and updates, can lead to optimal patient outcomes. Its alignment with global healthcare trends underscores its relevance and potential applicability in today's healthcare context, making the protocol a valuable asset to the scientific community.

SM & BM provided an equal contribution as first author in drafting the manuscript. All authors read and approved the final manuscript.

## Acknowledgments

We thank all the healthcare professionals, doctors and the management of the Dialysis Unit of the IRCCS Humanitas Rozzano, Milan

## Funding

This research did not receive any specific grant from funding agencies in the public, commercial, or not-for-profit sectors.

## CRediT authorship contribution statement

**Stefano Mancin:** Conceptualization, Methodology, Writing Original Draft, Review & Editing, Investigation, Visualization. **Beatrice Mazzoleni:** Conceptualization, Methodology, Writing Original Draft, Review & Editing, Investigation, Visualization. **Francesco Reggiani:** Review & Editing, Investigation, Visualization. **Marta Calatroni:** Review & Editing, Investigation, Visualization. **Elena Alterchi:** Review & Editing, Visualization, Coordinator. **Daniela Donizzetti:** Methodology, Review & Editing, Visualization, Coordinator. **Silvia Finazzi:** Conceptualization, Methodology, Review & Editing, Visualization; Coordinator. **Fanny Soekeland:** Review & Editing. **Marco Sguanci:** Writing Original Draft, Review & Editing, Visualization. **Salvatore Badalamenti:** Review & Editing, Visualization, Coordinator. Stefano Mancin and Beatrice Mazzoleni provided an equal contribution as first author in drafting the manuscript. All authors read and approved the final manuscript

## Declaration of Competing Interest

The authors declare that they have no known competing financial interests or personal relationships that could have appeared to influence the work reported in this paper.

## Data Availability

Data will be made available on request.

## Appendix Supplementary materials

Image, application 1
