# Supplementary material for: Integrated protocol for the prevention and treatment of skin ulcers in patients with end-stage renal disease
Source: MethodsX. 2023 Nov 10;11:102482. doi: 10.1016/j.mex.2023.102482 (PMC10689276; doi:10.1016/j.mex.2023.102482)
Supplement: Supplementary file 1 [file mmc1.docx]

**Supplementary material**

Upon request to the corresponding author, it will be possible to request educational brochures relating to the prevention of chronic ulcers.
